# Effects of growth under different light spectra on the subsequent high light tolerance in rose plants

**DOI:** 10.1093/aobpla/ply052

**Published:** 2018-09-12

**Authors:** Leyla Bayat, Mostafa Arab, Sasan Aliniaeifard, Mehdi Seif, Oksana Lastochkina, Tao Li

**Affiliations:** 1 Department of Horticulture, Aburaihan Campus, University of Tehran, Tehran, Iran; 2 Institute of Environment and Sustainable Development in Agriculture, Chinese Academy of Agricultural Science, Beijing, China; 3 Bashkir Research Institute of Agriculture, Russian Academy of Scienses, Ufa, Russia; 4 Institute of Biochemistry and Genetics, Russian Academy of Scienses, Ufa, Russia

**Keywords:** Antioxidant enzymes, high light, light spectrum, photosynthesis, pigments

## Abstract

Photosynthesis is defined as a light-dependent process; however, it is negatively influenced by high light (HL) intensities. To investigate whether the memory of growth under monochromatic or combinational lights can influence plant responses to HL, rose plants were grown under different light spectra [including red (R), blue (B), 70:30 % red:blue (RB) and white (W)] and were exposed to HL (1500 μmol m^−2^ s^−1^) for 12 h. Polyphasic chlorophyll a fluorescence (OJIP) transients revealed that although monochromatic R- and B-grown plants performed well under control conditions, the functionality of their electron transport system was more sensitive to HL than that of the RB- and W-grown plants. Before exposure to HL, the highest anthocyanin concentration was observed in R- and B-grown plants, while exposure to HL reduced anthocyanin concentration in both R- and B-grown plants. Ascorbate peroxidase and catalase activities decreased, while superoxide dismutase activity was increased after exposure to HL. This caused an increase in H_2_O_2_ concentration and malondialdehyde content following HL exposure. Soluble carbohydrates were decreased by exposure to HL, and this decrease was more emphasized in R- and B-grown plants. In conclusion, growing plants under monochromatic light reduced the plants ability to cope with HL stress.

## Teaser

Roses grew well under monochromatic light but were sensitive to subsequent high light stress. Growth under combinational light spectrum can improve resistance to subsequent high light stress. Carbohydrates help the plant to cope with high light stress. Anthocyanins degrade less in plants grown under combinational light, which helps the plant to cope with high light stress.

## Introduction

Light is the original source of energy for plant photosynthesis and growth. A wide range of signals and information for morphogenesis and many other physiological processes is triggered by light (Chen *et al.* 2004). Different characteristics of light such as spectral composition (wavelengths), intensity, duration and direction can influence plant growth and development. The photosynthesis process is also sensitive to all aspects of lighting environments.

The rapid development of lighting technologies using light-emitting diodes (LEDs) has caused an increase in the application of this technology for lighting in closed horticultural systems (Kozai *et al.* 2015). Light-emitting diodes are also attractive because of their high radiant efficiency, long lifetimes, small size, low temperature, narrow spectrum and physical robustness (Tennessen *et al.* 1994; Kim *et al.* 2004; Breive *et al.* 2005; Morrow 2008). The application of LEDs in horticulture makes it possible to use certain wavelengths to study particular plant responses. For instance, intercrop lighting using LEDs is nowadays used to promote photosynthesis of the middle and lower leaves. However, research on the effect of spectral wavelengths on plant growth and development is still in progress.

Plant responses to light differ based on the lighting environment, season, genotype, cultivation practices and many others (Kozai 2016). Although light is the energy source for photosynthesis, it can simultaneously function as a stress factor. Under high light (HL) intensity conditions or when plants are exposed to other abiotic stresses (e.g. drought), the energy supply (ATP) and reducing power (NADPH) through photosystems (and by involvement of electron transport chain) exceed the demand for metabolic processes in carbon-fixing reactions (Miyake *et al.* 2009; Gu *et al.* 2017). The accumulation of reactive oxygen species (ROS) is the result of a disturbance between supply and demand for electron transport end products. Depending on the rate of accumulation, ROS can have dual effects on plant responses. At low concentrations, ROS act as a signal to induce defence responses, while at high concentrations ROS are toxic and induce lipid peroxidation in the cell membranes and cause oxidative damage to cellular components (Dat *et al.* 2000; Vranová *et al.* 2002). Consequently, the functionality of photosynthesis decreases under stressful conditions. Photosynthesis suppression due to exposure to HL intensities is known as photoinhibition. Damage caused by the photoinhibition process can take place in all components of the photosynthetic machinery. Among them, the photosystem II (PSII) complex of the electron transport chain is considered the primary target of photoinhibition (Vass 2012; Roach and Krieger-Liszkay 2014). However, photosynthesis as a fine-tuned process employs multiple mechanisms to cope with photoinhibition. Dissipation of excess energy in the form of heat is the main protective strategy to get rid of the damage induced by HL intensities (Müller *et al.* 2001). Thermal dissipation is known as non-photochemical quenching (NPQ) of Chl fluorescence. Non-photochemical quenching encompasses several strategies including: energy-dependent quenching by involvement of the xanthophyll cycle, conformational changes in light harvesting complex II (LHCII), which are known as state transitions, and photoinhibition resulting in reduction of quantum yield as a consequence of light-induced damage (Müller *et al.* 2001; Zhao *et al.* 2017).

Light is absorbed by plant pigments. Chl a and b are the main photosynthetic pigments in plants. They mainly absorb blue and red wavelengths of the light spectrum. Carotenoids with an absorption spectrum between 350 and 500 nm are also found in all chlorophyll-based photosynthesis systems. They contribute to light absorption in the antenna system. Furthermore, carotenoids help the plant to dissipate excess energy as heat to protect the photosynthesis apparatus from HL intensities (Sharma and Hall 1993). Anthocyanins are also involved in the protection of photosynthetic apparatus from damage due to HL intensity (Petrella *et al.* 2016).

Roses are the most famous ornamental plants worldwide. The growth and development as well as post-harvest quality of rose plants are affected by pre-harvest environmental factors such as relative humidity (RH) and light (Fanourakis *et al.* 2013). Issues related to light have attracted much attention for rose cultivation during the last few decades. In some places (especially at high latitudes), supplementary lighting is used to compensate sun light limitations. In other places exposure to HL intensities during the summer negatively influences rose production. In those places shading the rose plants is a common practice during summer time. Furthermore, in many places plants are only exposed to HL conditions for a short duration. However, both shading and HL can negatively influence photosynthesis (García Victoria *et al.* 2011).

Each spectral band of light can induce certain responses in plants. For instance, red and blue lights mainly contain the range of wavelengths necessary for electron excitation in the photosynthetic apparatus (Taiz and Zeiger 2002). Wavelengths in the range of blue and UV cause the accumulation of carotenoids and anthocyanins in the leaves (Li and Kubota 2009; Carvalho *et al.* 2016), and red light induces accumulation of carbohydrates in the leaf (Sæbø *et al.* 1995). Since each band of light wavelengths can induce certain mechanisms and responses in plants, which can affect subsequent plant responses to environmental stresses, in the current study, we hypothesize that protective mechanisms against HL stress would be up-regulated/down-regulated through growing plants under certain light spectra which can influence plant responses to HL intensity afterwards. Furthermore, since limited wavelengths can induce certain protective mechanisms against HL, plants grown under combined wavelengths have more capabilities to tolerate HL intensities.

Cryptochromes and phototropins are the B light acceptors, whereas phytochromes are more sensitive acceptors for R light (Whitelam and Halliday 2008). These photoreceptors acquire information from the light environment and use that information to modulate cellular processes (Smith 1982). Therefore, different signal transduction pathways are involved in the up- or down-regulation of certain metabolic pathways by the information gained from specific photoreceptor of certain wavelength. The aims of the current study were to investigate (i) the structure and function of photosynthetic apparatus in plants (grown under different light spectra) exposed to HL intensity, and (ii) the mechanisms involved in HL tolerance in rose plants grown under different light spectra.

It has been shown that the energy flow and information related to the structure and function of the photosynthetic apparatus can be analysed through Chl fluorescence data. Although chlorophyll fluorescence parameters obtained by the saturation pulse method in light-adapted leaves give valuable information regarding the energy flow across thylakoid membranes, these types of measurement are time-consuming and are not fully ideal for assessing fluorescence parameters in practical conditions (Brestic and Zivcak 2013). The non-destructive analysis of polyphasic fast chlorophyll transient by the so-called OJIP test was developed for quick evaluation of biophysical aspects of photosynthesis especially under stress conditions (Strasser 1995; Mathur *et al.* 2013). This test relies on energy flow in thylakoid membranes, which provides detailed information about the biophysics of the photosynthetic system through measurement of fluorescence signals (Kalaji *et al.* 2017). The OJIP test has been successfully used for studying the photosynthetic apparatus of different plant species under abiotic stress conditions (Panda *et al.* 2008; Martinazzo *et al.* 2012; Kalaji *et al.* 2014; Rapacz *et al.* 2015; Kalaji *et al.* 2016). Therefore, this test was used in the current study to detect the negative effects of HL stress on the photosynthetic apparatus of the rose plants.

## Materials and Methods

### Plant material and growth conditions

**Figure 1.**
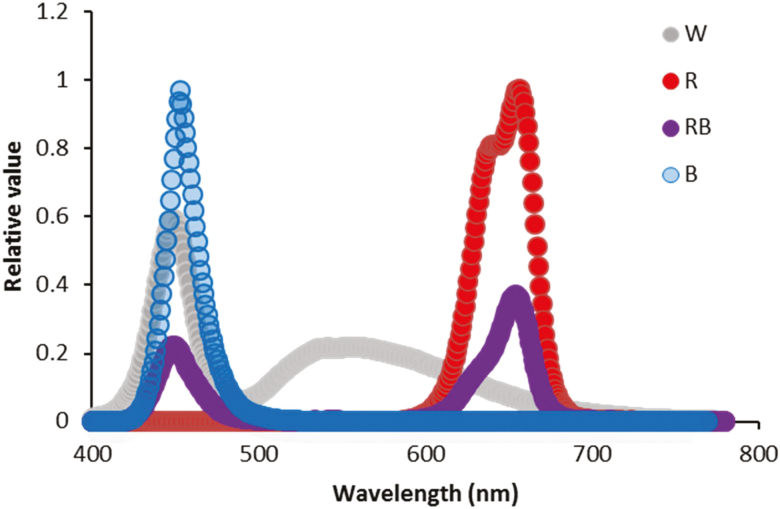
Light spectra of the blue (B), red (R), red and blue (RB) and white (W) lighting environments measured at plant level in the growth chambers.

**Figure 2.**
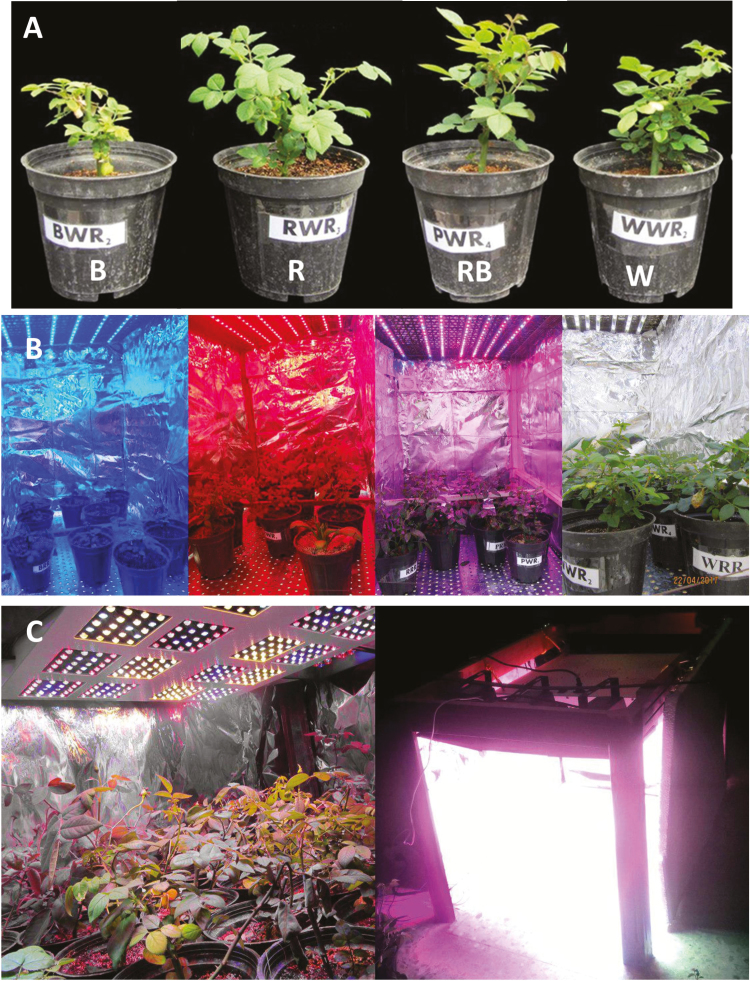
Representative images showing plants (A) that were grown for 3 weeks under different light spectra [blue (B), red (R), white (W) and red and blue (RB)], growth chambers that were used for growing plants under 250 (B) and 1500 (C) µmol m^−2^ s^−1^ PPFD.

Rose plants (*Rosa hybrida* cv. ‘Avalanche’) were propagated from cuttings in March 2017 in a perlite medium. Rooting was achieved following 4–5 weeks of planting. The rooted cuttings (10–15 cm long with two emerging leaves) were transplanted into 15 cm diameter plastic pots containing a mixture of cocopite and perlite (70/30 % by volume). The plants were watered daily for 3 days after transplantation, and thereafter, they were irrigated with half-strength Hoagland solution. Homogeneous plants were divided into four growth chambers (four plant per each growth chamber) with the same climatic conditions (temperature: 27 ± 3 °C; RH: 50 ± 5 %; photoperiod: 12-h light/12-h dark between 0800 and 2000 h). Plants were subjected to four different light spectra: white (W), blue (B), red (R) and 70 % red + 30 % blue (RB) provided by LED production modules (24-W, Iran Grow Light Co., Iran) at a photosynthetic photon flux density (PPFD) of 250 ± 10 μmol m^−2^ s^−1^. The W spectrum consisted of 41 % in the range of B and 18 % in the range of R. Photosynthetic photon flux density intensities and light spectra were monitored using a light meter (Sekonic C-7000, Japan). The relative spectra of the light treatments are shown in Fig. 1. Four weeks after growth at different light spectra, all plants were exposed to a HL intensity (1500 μmol m^−2^ s^−1^) for 12 h (between 0800 and 2000 h) (Fig. 2). To provide the same temperature during plant exposure to HL intensity as the growth chamber temperature, plants were illuminated with LED production modules (Iran Grow Light Co., Iran) in the range of 400–700 nm in a temperature-controlled room at 21 °C and 50 % RH. Under this condition, the temperature was fixed to 27 °C under HL intensity (same as the temperature in the growth chambers). Since such a HL intensity caused an increase in the temperature of growth chamber (27 °C), to maintain the same temperature before and after HL intensity same temperature was also kept inside the growth chambers during the growth of plants. The module consisted of 320 LEDs equipped with three fan ventilators to reduce the heat produced in the module. Measurement of the parameters was performed at two time points: first, at the end of plant growth under different light spectra with PPFD of 250 μmol m^−2^ s^−1^, and second, at the end of plant exposure to HL intensity (1500 μmol m^−2^ s^−1^).

### Chl fluorescence and OJIP test measurements

The youngest fully developed leaves were used for measuring the maximum quantum efficiency of PSII (*F*_v_/*F*_m_) with the Handy Fluorcam FC 1000-H (Photon Systems Instruments, PSI, Czech Republic). Intact leaves attached to the plants were dark-adapted for 20 min. After dark adaptation, intact plants were immediately used to measure *F*_v_/*F*_m_. The Fluorcam consisting of a CCD camera and four fixed LED panels, one pair supplying the measuring pulses and the second pair providing actinic illumination and saturating flash, were used. *F*_v_/*F*_m_ was calculated using a custom-made protocol (Genty *et al.* 1989; Aliniaeifard *et al.* 2014; Aliniaeifard and van Meeteren 2014). Images were recorded during short measuring flashes in darkness. At the end of the short flashes, the samples were exposed to a saturating light pulse (3900 µmol m^−2^ s^−1^) that resulted in a transitory saturation of photochemistry and reduction of the primary quinone acceptor of PSII (Genty *et al.* 1989). After reaching steady-state fluorescence, two successive series of fluorescence data were digitized and averaged, one during the short measuring flashes in darkness (*F*_0_), and the other during the saturating light flash (*F*_m_). From these two images, *F*_v_ was calculated by the expression *F*_v_ = *F*_m_ − *F*_0_. The *F*_v_/*F*_m_ was calculated using the ratio (*F*_m_ − *F*_0_)/*F*_m_. Maximum fluorescence (*F*_m_′) was determined in light-adapted steady state and was used for calculation of NPQ according to the following equation:

$$\text{NPQ}=(F_{\text{m}}/F_{\text{m}}{}^{\prime })$$ (1)

The average values and standard deviation per image were calculated using Fluorcam software version 7.0.

The polyphasic Chl a fluorescence (OJIP) transients were measured using a Fluorpen FP 100-MAX (Photon Systems Instruments, Drasov, Czech Republic) on young fully expanded rose leaves after 20 min dark adaptation. OJIP was used to study different biophysical and phenomenological parameters related to PSII status (Strasser 1995). The transient fluorescence measurement was induced by a saturating light of ~3000 μmol m^−2^ s^−1^. All the measurements were performed on 20 min dark-adapted plants. The OJIP transients were done according to the JIP test (Strasser *et al.* 2000b). The following data from the original measurements were used after extraction by Fluorpen software: fluorescence intensities at 50 μs (F50μs, considered as the minimum fluorescence *F*_0_), 2 ms (J-step, *F*_J_), 60 ms (I-step, *F*_I_) and maximum fluorescence (*F*_m_).

The performance index was calculated on the absorption basis (PI_ABS_) and densities of QA^−^ reducing PSII reaction centres at time 0 and time to reach maximum fluorescence. The yield ratios, including the probability that a trapped exciton moves an electron in the electron transport chain beyond QA^−^ (ψ_o_), the quantum yield of electron transport (φ_Eo_), the quantum yield of energy dissipation (φ_Do_) and the maximum quantum yield of primary photochemistry (φ_Po_), were also calculated based on the following equations:

$$V_{j}=(F_{j}-F_{0})/(F_{M}-F_{0})$$

$$\varphi_{Po}=1-\left(\frac{F_{0}}{F_{M}}\right)\;$$

$$\psi_{o}=1-V_{j}$$

$${\text{φ}}_{\text{Eo}}=\left(1-\left(\frac{F_{0}}{F_{M}}\right)\right)\cdot \;\psi_{o}$$

$$\varphi_{Do}=1-\varphi_{Po}-\left(\frac{F_{0}}{F_{M}}\right)$$

From these data the following parameters were calculated: the specific energy fluxes per reaction centre (RC) for energy absorption (ABS/RC = M_0_·(1/V_J_)·(1/φ_Po_)), (M_0_ = TR_0_/RC-ET_0_/RC), trapped energy flux (TR_0_/RC = M_0_· (1/V_J_)), electron transport flux (ET_0_/RC = M_0_·(1/V_J_)·ψ_o_) and dissipated energy flux (DI_0_/RC = (ABS/RC) – (TR_0_/RC)).

### Pigment measurements

The Chl and carotenoid contents of the leaves were measured according to the method described by Arnon (1949). For measuring the anthocyanin content of the leaves, 1 g of leaf tissue was homogenized in 10 mL methanol and the extract was incubated at 4 °C in the dark overnight. The slurry was centrifuged (SIGMA-3K30) at 4000 *g* for 10 min. The anthocyanin in the supernatant was spectrophotometrically (Lambda 25 UV/VIS spectrometer) determined at 520 nm according to the method described by Wagner (1979).

### Determination of hydrogen peroxide content and lipid peroxidation

The level of lipid peroxidation in the leaf tissue was measured based on the malondialdehyde (MDA, a product of lipid peroxidation) content of the leaves. Malondialdehyde was determined by the thiobarbituric acid (TBA) reaction by minor modification of the method described by Heath and Packer (1968). A 0.25 g leaf sample was homogenized in 5 mL of 0.1 % trichloroacetic acid (TCA). The homogenate was centrifuged at 10000 *g* for 5 min. Four millilitres of 20 % TCA containing 0.5 % TBA was added to a 1-mL aliquot of the supernatant. The mixture was heated at 95 °C for 30 min and then quickly cooled in an ice bath. After centrifuging at 10000 *g* for 10 min the absorbance of the supernatant at 532 nm was read and the value for the non-specific absorption at 600 nm was subtracted. The concentration of MDA was calculated using its extinction coefficient of 155 mM^−1^ cm^−1^ (Heath and Packer 1968).

Hydrogen peroxide (H_2_O_2_) content was spectrophotometrically measured after reaction with potassium iodide (KI). The reaction mixture contained 0.5 mL of 0.1 % TCA, leaf extract supernatant, 0.5 mL of 100 mm K-phosphate buffer and 2 mL reagent (1 M KI w/v in fresh double-distilled H_2_O). The blank contained 0.1 % TCA in the absence of leaf extract. The reaction was developed for 1 h in darkness and absorbance was measured at 390 nm. The amount of H_2_O_2_ was calculated using a standard curve prepared with known concentrations of H_2_O_2_ according to the method described by Patterson *et al.* (1984).

### Determination of carbohydrates

Young fully developed leaves (300 mg fresh weight [FW]) were collected from each replicate per treatment and were ground in liquid nitrogen, mixed with 7 mL of 70 % ethanol (w/v) for 5 min on ice and centrifuged at 6700 *g* for 10 min at 4 °C. After adding 200 mL of the supernatant to 1 mL of an anthrone solution (0.5 g anthrone, 250 mL 95 % H_2_SO_4_ and 12.5 mL distilled water), the absorbance was spectrophotometrically recorded at 625 nm (van Doorn 2012).

For starch quantification, 0.1 g of a fresh fully developed leaf was ground and sugars were extracted with 80 % ethanol and starch was solubilized using 52 % perchloric acid. Starch was colorimetrically determined at 630 nm using the sugar-anthrone-sulfuric acid reaction based on the method described by McCready *et al.* (1950).

### Determination of activities of antioxidant enzymes

Ascorbate peroxidase (APX) activity was determined by oxidation of ascorbic acid (AA) at 265 nm (ε = 13.7 mM^−1^ cm^−1^) by slight modification of the method described by Nakano and Asada (1981). The reaction mixture contained 50 mM potassium phosphate buffer (pH 7.0), 5 mM AA, 0.5 mM H_2_O_2_ and the enzyme extract. The reaction was started by adding H_2_O_2_. The rates were corrected for the non-enzymatic oxidation of AA by the inclusion of a reaction mixture without the enzyme extract (blind sample). The enzyme activity was expressed in μmol AA min^−1^ per g of fresh weight (Nakano and Asada 1981).

For determination of catalase (CAT) activity, the decomposition of H_2_O_2_ was recorded by the decrease in absorbance at 240 nm. Reaction mixture consisted of 1.5 mL 50 mM sodium phosphate buffer (pH 7.8), 0.3 mL 100 mM H_2_O_2_ and 0.2 mL enzyme extract. One CAT unit was defined as the amount of enzyme necessary to decompose 1 mM min^−1^ H_2_O_2_. Therefore, the CAT activity was expressed as U g^−1^ FW min^−1^ (Díaz-Vivancos *et al.* 2008).

Superoxide dismutase (SOD) activity was measured according to the method described by Dhindsa *et al.* (1981). The method is based on the ability of SOD to inhibit the photochemical reduction of nitro blue tetrazolium (NBT). The reaction mixture contained 50 mM phosphate buffer (pH 7–8), 13 mM methionine, 75 µM NBT, 0.15 mM riboflavin, 0.1 mM EDTA and 0.50 mL enzyme extract. Riboflavin was the last item that was added. The reaction mixture was shaken and placed under two 15-W fluorescent lamps. The reaction was started by switching on the light and was allowed to run for 10 min. The reaction was stopped by turning off the light and the tubes were covered using a black cloth. The absorbance by the reaction mixture was spectrophotometrically recorded at 560 nm. A reaction mixture without exposure to the light was used as the control. No colour was observed in the control. Under assay conditions, one unit of SOD activity is expressed as the amount of enzyme that led to 50 % inhibition of NBT reduction (Dhindsa *et al.* 1981).

### Statistical analysis

Four plants were used as four replicates in each light treatment. Individual plants were taken as independent replicates. The measurements were done on two timescales: before and after exposure to HL. To make the environmental conditions of the two timescales similar, environmental conditions inside the chambers and between the chambers were kept as similar as possible before and after exposure to HL. To do this, the same temperature, RH and light intensity and duration were maintained during these two timescales in a climate-controlled room. Sampling was done on the same plants before and after exposure to HL. The data were subjected to two-way analysis of variance (ANOVA) and the Tukey test was used as a post-test. *P* > 0.05 was considered not significant. GraphPad Prism 7.01 for Windows (GraphPad Software, Inc., San Diego, CA) was used for the statistical analysis.

## Results

### Polyphasic Chl a fluorescence (OJIP) transients

**Figure 3.**
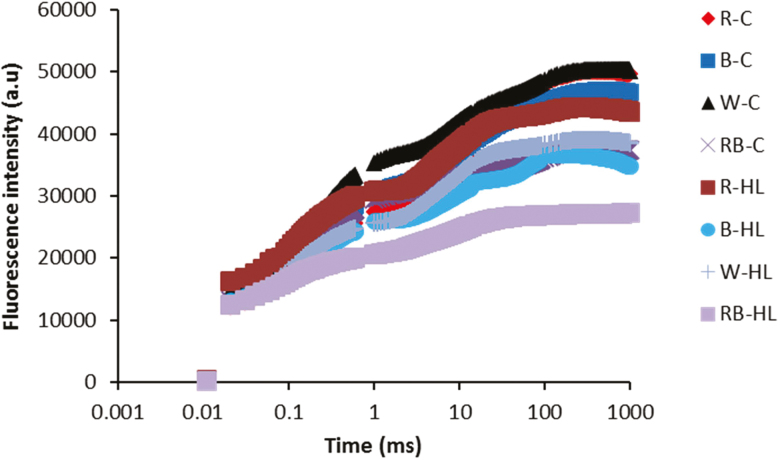
Fast chlorophyll fluorescence induction curve exhibited by leaves of rose plants exposed to different light spectra [blue (B), red (R), white (W) and red and blue (RB)] under 250 (C) and 1500 (HL) µmol m^−2^ s^−1^ PPFD.

In the current study, the PSII activity was studied by calculation of different Chl fluorescence parameters in a leaf in the dark-adapted state. Investigating the fast chlorophyll fluorescence induction curve showed that all plants produced typical polyphasic curves with the basic OJIP steps (Fig. 3). For all plants, the intensity of the fluorescence signal was increased from the initial fluorescence level (*F*_0_) to the intermediate steps (J and I) and then reached the maximum level (*F*_m_). Except for R light, exposure to HL caused a decline in fluorescence intensity in all steps of OJIP.

**Figure 4.**
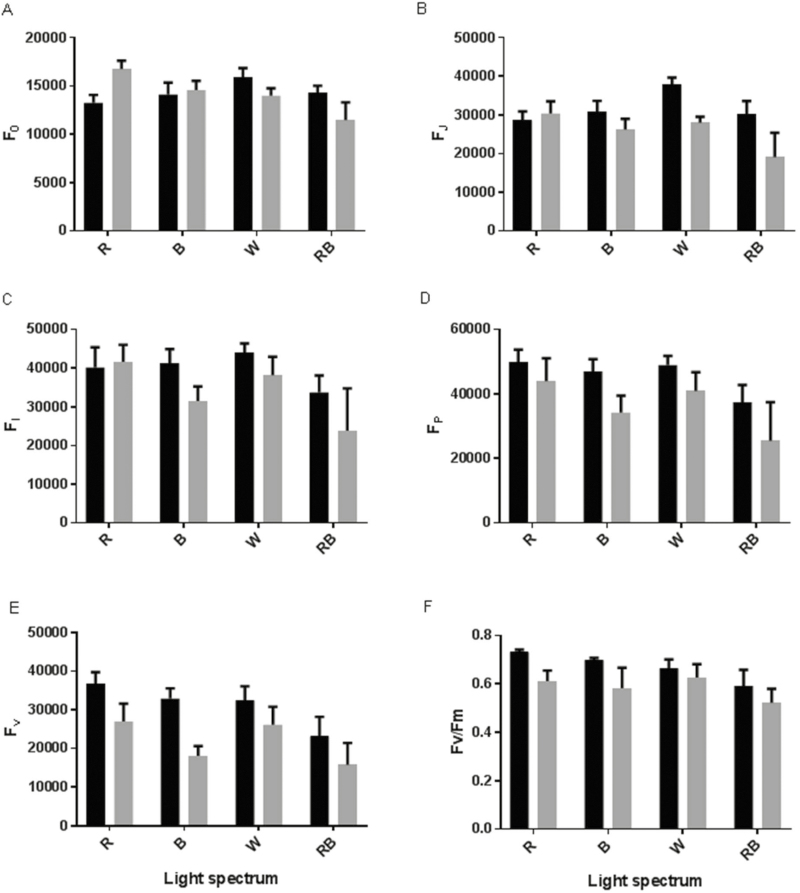
Intensity of chlorophyll a fluorescence in the OJIP test including *F*_0_ (A), *F*_J_ (B), *F*_I_ (C), *F*_P_ (D), *F*_v_ [E; (*F*_m_ − *F*_0_)] and *F*_v_/*F*_m_ (F) from the fluorescence transient exhibited by leaves of rose plants grown under different light spectra [blue (B), red (R), white (W) and red and blue (RB)] under 250 (black bars) and 1500 (grey bars) µmol m^−2^ s^−1^ PPFD. Bars represent means ± SD.

**Figure 5.**
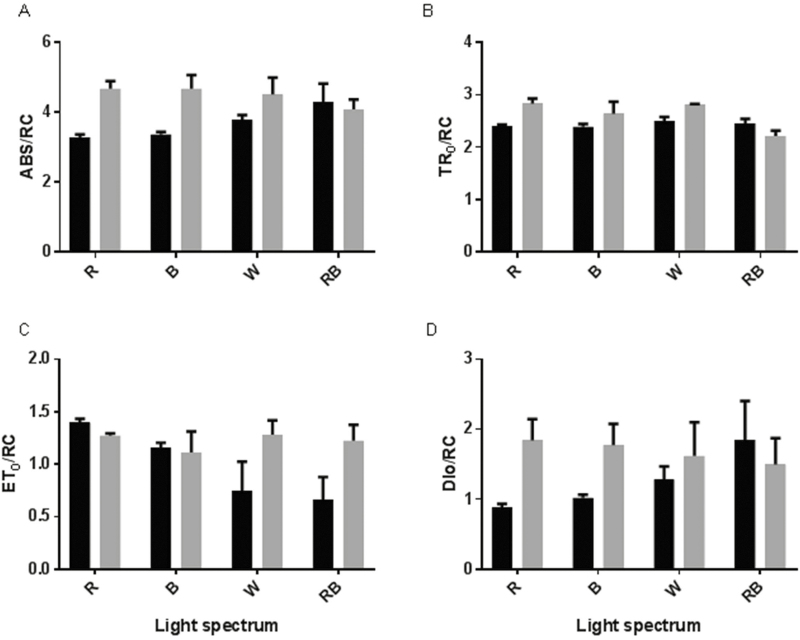
Specific energy fluxes per reaction centre (RC) for energy absorption [A; (ABS/RC)], trapped energy flux [B; (TR_0_/RC)], electron transport flux [C; (ET_0_/RC)] and dissipated energy flux [D; (DI_0_/RC)] from the fluorescence transient exhibited by leaves of rose plants grown under different light spectra [blue (B), red (R), white (W) and red and blue (RB)] under 250 (black bars) and 1500 (grey bars) µmol m^−2^ s^−1^ PPFD. Bars represent means ± SD.

Under control (C) conditions, the highest *F*_0_ and *F*_J_ were observed for W-grown plants and the lowest values were detected in R-grown plants (Fig. 4A and B). Under HL conditions, the highest *F*_0_, *F*_J_, *F*_I_ and *F*_P_ were observed for R-grown plants and their lowest values were detected in RB-grown plants (Fig. 4A–D). In all plants, exposure to HL resulted in a decrease in *F*_v_ values; however, among the light treatments, the change in *F*_v_ following exposure to HL was the lowest for W-grown plants (Fig. 4E). *F*_v_/*F*_m_ also decreased following exposure to HL and this decrease was greatest for monochromatic R- and B-grown plants (Fig. 4D). The calculated parameters of the OJIP test were changed significantly due to exposure to different light spectra and HL, and the interactions were also significant for the calculated parameters **[see****Supporting Information—Table S1****]**. The calculated parameters for specific energy fluxes per reaction centre such as ABS/RC, TR_0_/RC and DI_0_/RC were increased following exposure to HL for all plants grown under different light spectra, except for the RB-grown plants (Fig. 5A, B and D). In the case of ABS/RC and DI_0_/RC, the highest difference between C and HL was observed in monochromatic R- and B-grown plants. There was no significant difference between C and HL for the RB-grown plants. ET_0_/RC was similar in the C and HL treatments for R- and B-grown plants, while it was increased in W- and RB-grown plants after exposure to HL (Fig. 5C).

**Figure 6.**
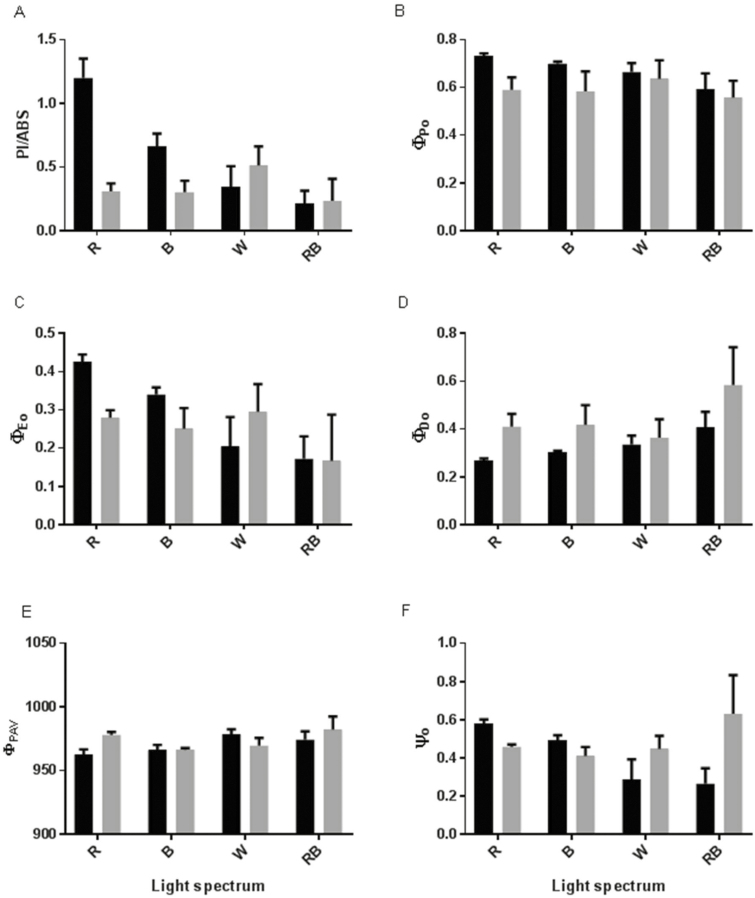
Performance index on the absorption basis [A; (PI_ABS_)], maximum quantum yield of primary photochemistry [B; (φ_Po_)], quantum yield of electron transport [C; (φ_Eo_)], quantum yield of energy dissipation [D; (φ_Do_)], average (from time 0 to *t*_FM_) quantum yield for primary photochemistry [E; (φ_PAV_)] and the probability that a trapped exciton moves an electron in the electron transport chain beyond QA^−^ [F; (ψ_o_)] from the fluorescence transient exhibited by leaves of rose plants grown under different light spectra [blue (B), red (R), white (W) and red and blue (RB)] under 250 (black bars) and 1500 (grey bars) µmol m^−2^ s^−1^ PPFD. Bars represent means ± SD.

Analysis of the parameters that estimate the yields and efficiency of the electron transport chain showed that although the highest values for PI_ABS_, φ_Po_ and φ_Eo_ were observed in monochromatic R- and B-grown plants under control conditions, their values were decreased more in the HL condition in comparison with their values in W- and RB-grown plants (Fig. 6A–C). Exposure to HL led to an increase in φ_Do_ and NPQ **[see****Supporting Information—Fig. S1****]** in all plants; however, the highest φ_Do_ was observed in RB-grown plants under both control and HL conditions (Fig. 6D). An increase in φ_RAV_ was observed in R- and RB-grown plants following exposure to HL (Fig. 6E). R- and RB-grown plants had the highest ψ_o_ values under control conditions, while their ψ_o_ values decreased following exposure to HL. Conversely, in W- and RB-grown plants ψ_o_ values were low under control conditions and were increased after exposure to HL (Fig. 6F).

### Pigments

**Figure 7.**
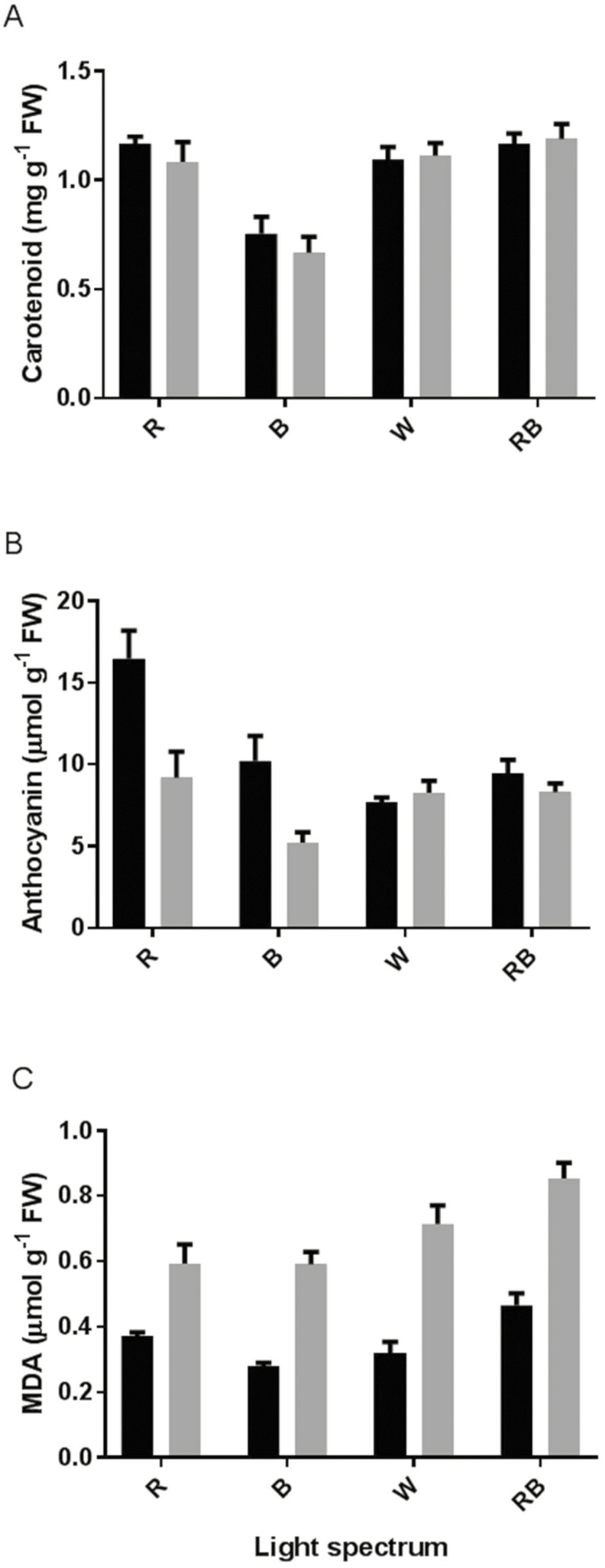
Carotenoid (A) and anthocyanin (B) concentrations and MDA (C) content in the leaves of rose plants grown under different light spectra [blue (B), red (R), white (W) and red and blue (RB)] under 250 (black bars) and 1500 (grey bars) µmol m^−2^ s^−1^ PPFD. Bars represent means ± SD.

All photosynthetic pigments were significantly influenced by the light spectra (Fig. 7). Chl a **[see****Supporting Information—Fig. S2A****]**, b **[see****Supporting Information—Fig. S2B****]** and total Chl **[see****Supporting Information—Fig. S2C****]** as well as carotenoids (Fig. 7A) were significantly decreased by growing rose plants under blue light. In the case of anthocyanin, the interaction between light spectra and light intensity was significant (*P* ≤ 0.01). Before exposure to HL, the highest anthocyanin concentration was observed in R- and B-grown plants, while exposure to HL led to an ~50 % reduction in anthocyanin concentration in both R- and B-grown plants; significant differences were not found for anthocyanin concentration before and after HL in the leaves of W- and RB-grown plants (Fig. 7B).

### Antioxidant enzymes and oxidative damage

**Figure 8.**
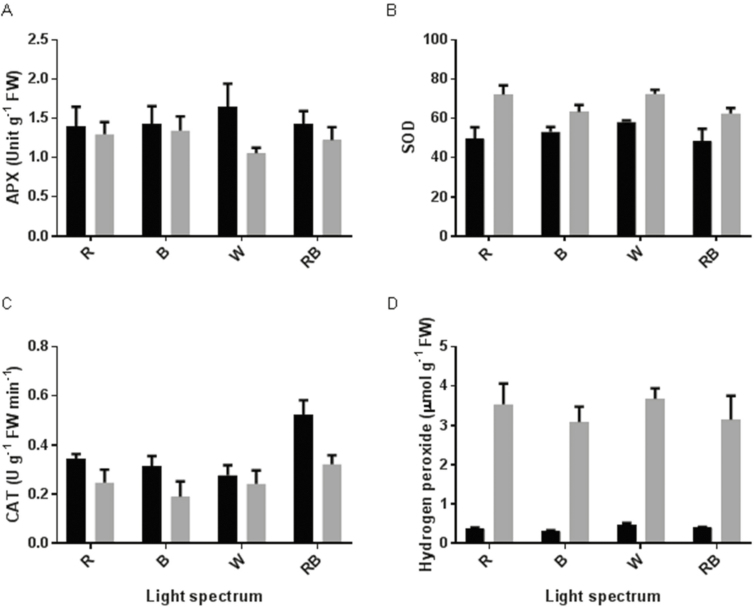
Activity of APX (A), SOD (B) and CAT (C) and H_2_O_2_ concentration in the leaves of rose plants grown under different light spectra [blue (B), red (R), white (W) and red and blue (RB)] under 250 (black bars) and 1500 (grey bars) µmol m^−2^ s^−1^ PPFD. Bars represent means ± SD.

Ascorbate peroxidase and SOD were significantly influenced by light intensity in contrasting ways (Fig. 8A and B). Irrespective of the light spectra, APX activity was reduced following exposure to HL, while SOD activity was induced by HL exposure. Catalase activity was reduced by exposure to HL (Fig. 8C). The highest CAT activity was observed in RB-grown plants in both control and HL conditions. The H_2_O_2_ level was considerably influenced by light intensity (Fig. 8D). In comparison with control conditions, the H_2_O_2_ level was increased by 10 times following exposure to HL. Malondialdehyde content was also influenced by light intensity (Fig. 7C). In comparison with control conditions, the MDA content was doubled after exposure to HL. The highest MDA content was observed in RB-grown plants in both control and HL conditions.

### Carbohydrate concentrations

**Figure 9.**
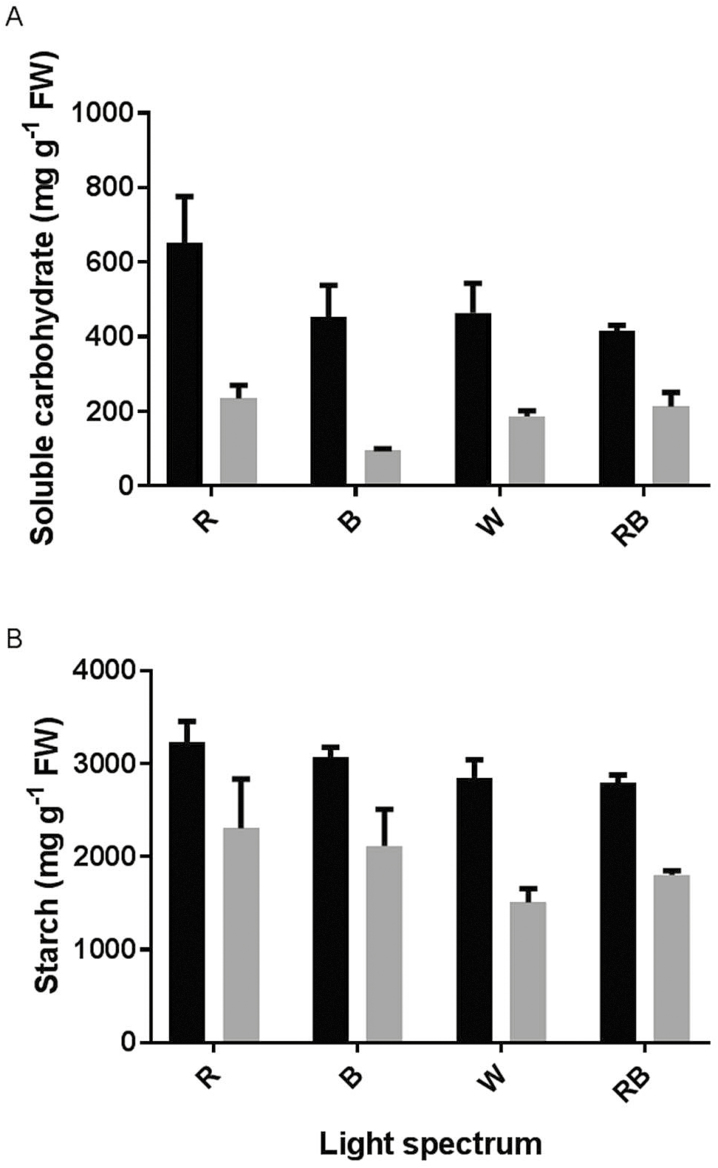
Concentration of soluble carbohydrate (A) and starch (B) in the leaves of rose plants grown under different light spectra [blue (B), red (R), white (W) and red and blue (RB)] under 250 (black bars) and 1500 (grey bars) µmol m^−2^ s^−1^ PPFD. Bars represent means ± SD.

Soluble carbohydrates were considerably decreased by exposure to HL conditions (Fig. 9A). This decrease was greater in R- and B-grown plants (65 % and 79 % decrease, respectively) than in W- and RB-grown plants (60 % and 49 % decrease, respectively). The highest and lowest concentrations of soluble carbohydrates were detected in R- and B-grown plants under control and HL conditions, respectively.

Similarly to soluble carbohydrates, HL conditions led to a decrease in starch concentration (Fig. 9B). This decrease was, respectively, 28 % and 31 % for R- and B-grown plants and 46 % and 35 % for W- and RB-grown plants. The highest and lowest concentrations of starch were, respectively, detected in R- and W-grown plants under both control and HL conditions.

## Discussion

The reactions involved in photosynthesis in plants are dependent on environmental conditions. Different light attributes including spectrum and intensity are considered to have the most direct effects on photosynthetic reactions (Chen *et al.* 2004). In our study, Chl fluorescence data were analysed using the OJIP test. Using this test we showed that the PSII system appropriately operated in plants grown under monochromatic R and B spectra. However, when R- and B-grown plants were exposed to HL conditions, their PI_ABS_ and ϕ_Eo_ decreased and DI_0_/RC increased in comparison with their values under control conditions (Figs 5–7). On the other hand, in W- and RB-grown plants ψ_o_ and ET_0_/RC increased following exposure to HL (Figs 5 and 7). These results revealed that growing plants under combination of light spectra enabled the plants to develop a photosynthesis apparatus with lower vulnerability than the photosynthesis apparatus in monochromatically grown plants in response to HL stress. The PI_ABS_ amalgamates the energy fluxes from the early step of the absorption process until the plastoquinone reduction (Strasser *et al.* 2000a). According to previous reports this parameter is very sensitive to different environmental stresses and has been successfully used to measure photosynthetic and plant performance in response to different abiotic stresses including high temperatures (Martinazzo *et al.* 2012), salinity (Mathur *et al.* 2013), nutrient deficiency (Kalaji *et al.* 2014), submergence (Sarkar and Ray 2016), cold (Rabara *et al.* 2017) and low pH (Long *et al.* 2017).

In the current experiment, to ensure the same temperature before and after HL, same temperature (27 °C) was also kept inside the growth chambers, which negatively influenced the Chl a fluorescence parameters, resulting in values slightly lower than 0.8 for *F*_v_/*F*_m_ (Fig. 4). The lower PSII photochemical performance (lower PI_ABS_ and φ_Eo_) in monochromatic R- and B-grown plants following exposure to HL was due to the higher light energy absorption (ABS/RC), trapping (TR_0_/RC) and dissipated energy flux (DI_0_/RC) and lower ET_0_/ABS per reaction centre (Fig. 5), which consequently resulted in a decreased quantum yield of electron transport (φ_Eo_) and maximum quantum yield of primary photochemistry (φ_Po_) (Fig. 6). The increase in ABS/RC could attribute to the inactivation of reaction centres and a decrease in active QA reducing centres (Strasser and Stirbet 1998). Under HL conditions, defective QA reducing centres function as a heat sink and protect the plant from HL damage (Zivcak *et al.* 2014). The higher value of TR_0_/RC resulted in higher inhibition of reoxidation of QA^–^ to QA (Strasser *et al.* 2000a). Higher TR_0_/RC would result in lower electron transport per reaction centre (ET_0_/RC) (Fig. 5), in turn resulting in reduced electron transport per trapping. In fact, a low proportion of absorbed energy is conveyed on the electron transport chain (Sarkar and Ray 2016). Limitation of electron transport beyond PSII will result in QA over-reduction. High light restricts electron transport towards the cytb6f complex and causes QA over-reduction (Foyer *et al.* 2012). In W- and RB-grown plants, electron transport flux per reaction centre and the probability that a trapped exciton would move an electron in the electron transport chain beyond QA^−^ (ψ_o_) increased following HL stress (Figs 6 and 7). This showed that W- and RB-grown plants were more capable of transporting electrons from absorbed photons into the electron transport chain and beyond QA^−1^. This confirmed that W- and RB-grown plants were positively regulating the energy level in reaction centres (Strasser *et al.* 2004) following exposure to HL.

Plants’ adaptation to the prevailing light environment is reflected by a change in anatomy and morphology of leaves, which is known as developmental acclimation (Vialet-Chabrand *et al.* 2017). Rapid and short-term exposure to HL intensities (as happened in the current study) results in the dynamic acclimation of plants to the light environment (Lawson *et al.* 2012). Dynamic responses to HL intensities involve metabolic modifications, the down-regulation of the electron transport chain, and changes in stomatal responses and the activation rate of the a (Tinoco-Ojanguren and Pearcy 1995; Lawson *et al.* 2012). In the current study, the focus was on dynamic responses to HL intensities. Therefore, metabolic modifications and changes in performance of the electron transport system were studied in details. Down-regulation of the electron transport system was an obvious response of the photosynthesis system to HL conditions. The antenna in association with PSII is a highly dynamic complex; it collects and transfers the light energy to the PSII reaction centre; however, depending on the physiological needs of the plant, it has the ability to decrease the amount of deliverable light energy to tune the excitation of the reaction centre (Horton *et al.* 1996). To cope with HL stress, plants dissipate excess energy in the form of heat by a process known as NPQ of chlorophyll fluorescence. Using NPQ, the excitation energy of chlorophyll molecules in the antennae is not passed to the reaction centre and will be dissipated as heat (Krause and Weis 1991). Consistent with this, in the current study, exposure to HL conditions resulted in an increase in NPQ **[see****Supporting Information—Fig. S1****]**.

**Figure 10.**
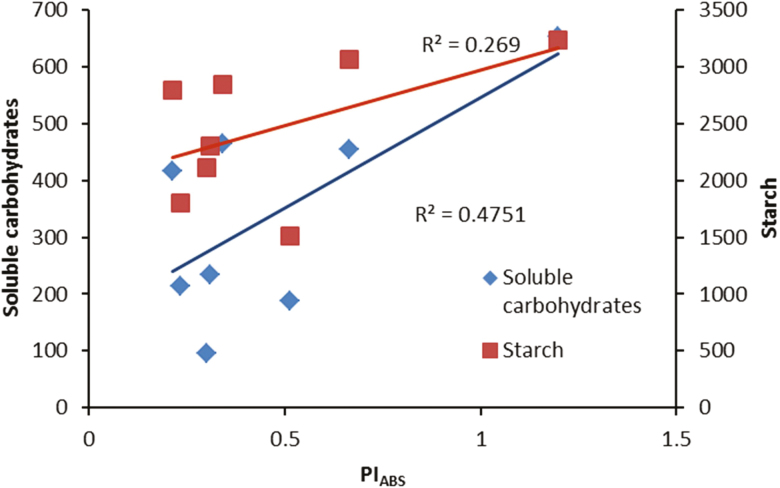
Relationship between performance index on the absorption basis (PI_ABS_) and concentration of carbohydrates (soluble carbohydrates and starch) in the leaves of rose plants grown under different light spectra [blue (B), red (R), white (W) and red and blue (RB)] under 250 (C) and 1500 (HL) µmol m^−2^ s^−1^ PPFD.

Metabolic modifications due to HL were studied by analysing carbohydrate and pigment concentrations at the same time as analysing components of oxidative stress. Soluble carbohydrates and starch concentrations were dramatically decreased after exposure to HL conditions (Fig. 9). Although light is the driving force of photosynthesis for the production of carbohydrates, our result showed that in plants grown under illumination of 250 μmol m^−2^ s^−1^ subsequent exposure to HL conditions (1500 μmol m^−2^ s^−1^) dramatically reduced both soluble and storage carbohydrates (Fig. 9). Positive relationships were found between both soluble carbohydrates and starch with PI_ABS_ (Fig. 10). This indicates that the performance of the PSII operating system plays an important role in soluble carbohydrate and starch production in the rose plant. The greater decrease in soluble carbohydrates in R- and B-grown plants following HL stress is indicative of the higher sensitivity of their PSII operating system to HL stress.

**Figure 11.**
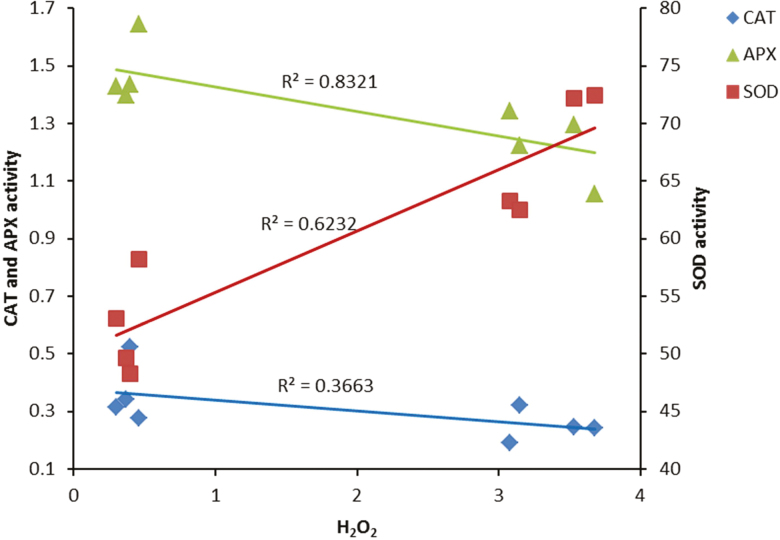
Relationship between H_2_O_2_ concentration and activity of antioxidant enzymes (CAT, APX and SOD) in the leaves of rose plants grown under different light spectra [blue (B), red (R), white (W) and red and blue (RB)] under 250 (C) and 1500 (HL) µmol m^−2^ s^−1^ PPFD.

In our study, H_2_O_2_ accumulation was detected following HL stress (Fig. 8). The production of ROS following exposure to various environmental stress conditions has previously been reported (Steffens 2014; Pospíšil 2016). In photosynthetic electron transport systems, excess light energy beyond photosynthetic capacity induces the production of ROS (Pospíšil 2016). Non-enzymatic and enzymatic scavenging systems are involved in the removal of ROS formed in the electron transport system during exposure to stress conditions (Ahmad *et al.* 2010). During HL stress, the balance between the scavenging system and ROS formation can be disturbed, leading to oxidative damage to the PSII operating system and its components (Aro *et al.* 1993). Moreover, in such circumstances, the excess energy absorbed by the PSII complex is not coupled with electron transport, which results in full reduction of the PQ pool and blockage of the electron (Pospíšil 2016). To decrease the reduction of the PQ pool, an electron from Q_A_^•−^ is transported to an O_2_ molecule and generates a superoxide anion. A superoxide anion radical is also generated during the Mehler reaction at the acceptor side of photosystem I. This radical is converted by SOD to H_2_O_2_ and subsequently to H_2_O by CAT and APX (Caverzan *et al.* 2012). We showed that in contrast with a positive correlation between SOD activity and H_2_O_2_ production (Fig. 11), CAT and APX negatively regulate H_2_O_2_ production. The accumulation of H_2_O_2_ following HL stress could be attributed to an increase in SOD activity and decrease in CAT and APX activities (Fig. 8). Photoinactivation of CAT by HL intensities has previously been reported. In accordance with our results, Feierabend and colleagues (1996) showed that CAT is photoinactivated when exposed to B or R lights.

**Figure 12.**
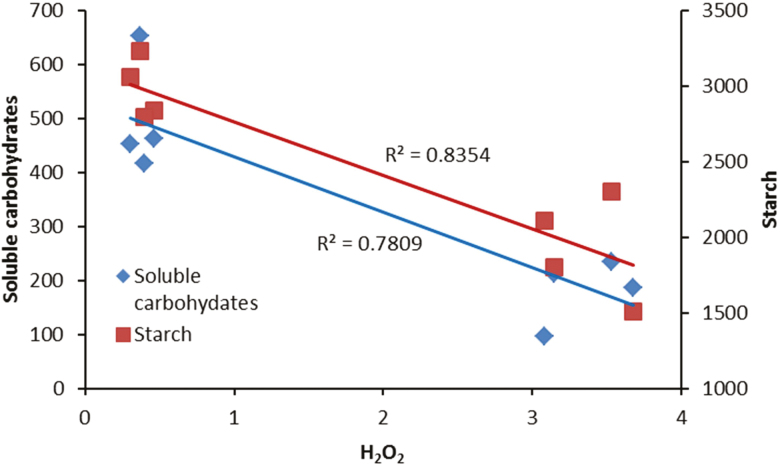
Relationship between H_2_O_2_ concentration and concentration of carbohydrates (soluble carbohydrates and starch) in the leaves of rose plants grown under different light spectra [blue (B), red (R), white (W) and red and blue (RB)] under 250 (C) and 1500 (HL) µmol m^−2^ s^−1^ PPFD.

In our study, negative relationships were discovered between carbohydrates (soluble carbohydrates and starch) and H_2_O_2_ concentration (Fig. 12). In previous studies, it has been reported that sugar accumulation occurs when plants are grown under high illuminations. The relationship between sugars and ROS accumulation is not just a simple positive relationship. Both acceleration of certain ROS-production pathways and deceleration of other ROS-production pathways have been reported due to high levels of soluble carbohydrates (Couée *et al.* 2006).

**Figure 13.**
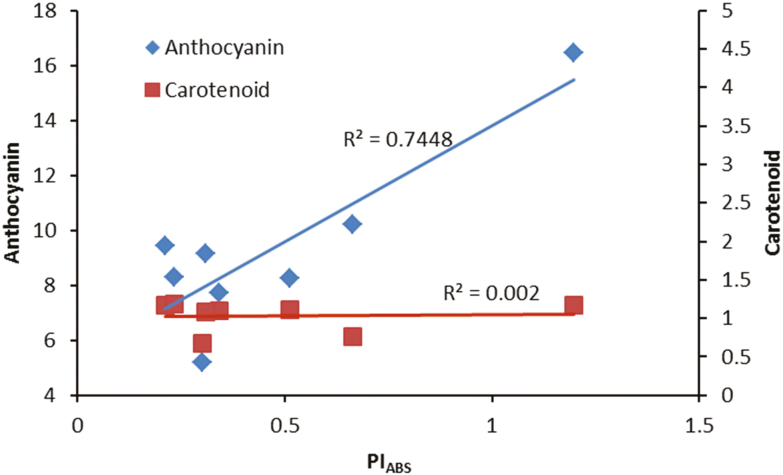
Relationship between performance index on the absorption basis (PI_ABS_) and pigments (anthocyanin and carotenoid) in the leaves of rose plants grown under different light spectra [blue (B), red (R), white (W) and red and blue (RB)] under 250 (C) and 1500 (HL) µmol m^−2^ s^−1^ PPFD.

We found a positive relationship between anthocyanin concentrations and PI_ABS_ under our experimental set-up (Fig. 13). Our data revealed that although plants grown under monochromatic R and B lights had higher anthocyanin concentrations which resulted in better performance of their PSII operating system, their anthocyanin concentrations were dramatically decreased following exposure to HL stress and as a result their PI_ABS_ were also decreased accordingly. In contrast, anthocyanin concentrations in W- and RB-grown plants were not changed before or after HL stress. Therefore, anthocyanin protects the photosynthetic apparatus against HL stress. Apparently, the negative relationship between soluble carbohydrates and ROS accumulation is dependent on the type and duration of the stress condition. For instance, exposure to low temperatures under normal to high irradiances would result in photo-oxidative damage (Harvaux and Kloppstech 2001) and accumulation of soluble carbohydrates. Under such conditions, sugar accumulation can help plants to cope with HL damage (Ciereszko *et al.* 2001). Carbohydrate accumulation can induce the production of anthocyanin. Chalcone synthase as the key enzyme in anthocyanin biosynthesis can be activated by the signal from soluble carbohydrates (Koch 1996; Deng *et al.* 2014). Therefore, HL stress can induce the accumulation of both anthocyanins and ROS (Steyn *et al.* 2002; Hatier and Gould 2008). In our study, the level of soluble carbohydrates and anthocyanins decreased following exposure to HL to both monochromatic lights, while the levels of these reductions were lower in W- and RB-grown plants. As a consequence, there was lower damage to their PSII operating system (Figs 10 and 13). Anthocyanins can act as light filters and protect the photosynthetic components against photoinhibition induced by HL stress (Landi *et al.* 2015).

## Conclusions

The management of a favourable lighting environment for rose production has attracted much attention in the last decade. Currently, many research teams all over the world are focusing on issues related to the best light intensity and spectra for rose plants. To prevent HL intensities shading screens are used for rose production. However, both shading and HL can negatively influence plant growth and photosynthesis. Using polyphasic chlorophyll a fluorescence transients (OJIP test), we showed that growing plants under monochromatic R and B lights resulted in higher sensitivity of their PSII operating system to HL compared to the PSII performance of RB- and W-grown plants. Superoxide dismutase activity increased while CAT and APX activity decreased after exposure to HL. This resulted in the accumulation of H_2_O_2_ following HL exposure. Negative relationships were discovered between carbohydrates (soluble carbohydrates and starch) and H_2_O_2_ concentration, while carbohydrate concentration positively influenced the performance index of the plants. Our study revealed the protective role of anthocyanin for the PSII operating system. The performance capacity of the photosynthetic systems in monochromatic R- and B-grown plants declined due to a dramatic reduction in anthocyanin concentration following HL exposure, whereas W- and RB-grown plants had similar anthocyanin levels before and after HL exposure. This suggests that anthocyanin protected their photosynthetic apparatus against HL stress.

## Sources of Funding

We would like to thank Iran National Science Foundation (INSF) (grant number 96006991) and University of Tehran for their supports.

## Contributions by the Authors

S.A. made substantial contributions to conception and design, also performed statistical analysis, drafted the manuscript and critically revised the final version. L.B. carried out the experiments, collected and critically analysed the scientific literatures and help in the writing of the manuscript. M.A. took part in designing and planning the experiments, preparation of material for the experiment and contributed to scientific discussion of the obtained results. M.S. helped in preparation of material for the experiment and took part in designing, planning and performing of experiments. T.L. contributed to conception and design of experiment, preparation of materials, scientific discussion and critical revision of the final manuscript. O.L. contributed to design of experiment and critical revision of the final manuscript.

## Conflict of Interest

None declared.

## Supporting Information

The following additional information is available in the online version of this article—

**Table S1.** Analysis of variance (*F*-values) for assessed parameters for rose plants grown under different light spectrums and then exposed to high light intensity (1500 μmol m^−2^ s^−1^).

**Figure S1.** Non-photochemical quenching (NPQ) derived from chlorophyll fluorescence parameters in the leaves of rose plants grown at different light spectrums [blue (B), red (R), white (W) and red and blue (RB)] under 250 (black bars) and 1500 (grey bars) µmol m^−2^ s^−1^ photosynthetic photon flux density (PPFD). Bars represent means ± SD.

**Figure S2.** Chlorophyll a (A), chlorophyll b (B) and total chlorophyll (C) concentrations in the leaves of rose plants grown at different light spectrums [blue (B), red (R), white (W) and red and blue (RB)] under 250 (black bars) and 1500 (grey bars) µmol m^−2^ s^−1^ photosynthetic photon flux density (PPFD). Bars represent means ± SD.

Supporting Information
