# Supplementary material for: Effects of growth under different light spectra on the subsequent high light tolerance in rose plants
Source: AoB Plants. 2018 Sep 12;10(5):ply052. doi: 10.1093/aobpla/ply052 (PMC6191502; doi:10.1093/aobpla/ply052)
Supplement: Supporting Information [file ply052_suppl_supporting_information.docx]

**Supporting Information**

**Table S1.** Analysis of variance (*F* values) for assessed parameters for rose plants grown under different light spectrums and then exposed to high light intensity (1500 μmol m^-2^ s^-1^).

|  | Independent variables | | |
| --- | --- | --- | --- |
| Dependent Variable | Light  spectra | Light intensity | Interaction  (spectra×intensity) |
| F_0_ | 0.17 ^ns^ | 0.79 ^ns^ | 0.03^*^ |
| F_J_ | 0.11 ^ns^ | 0.02 ^*^ | 0.19 ^ns^ |
| F_I_ | 0.05^*^ | 0.11 ^ns^ | 0.68 ^ns^ |
| F_p_ | 0.05 ^*^ | 0.03 ^*^ | 0.92 ^ns^ |
| F_v_ | 0.03^*^ | 0.01^**^ | 0.73 ^ns^ |
| F_v_/F_m_ | 0.16 ^ns^ | 0.02^*^ | 0.82 ^ns^ |
| PI_ABS_ | 0.01^**^ | 0.01^**^ | 0.01^**^ |
| ABS/RC | 0.8 ^ns^ | 0.01^**^ | 0.05^*^ |
| TR_0_/RC | 0.02^*^ | 0.02^*^ | 0.02^*^ |
| ET_0_/RC | 0.1 ^ns^ | 0.05^*^ | 0.05^*^ |
| DI_0_/RC | 0.7 ^ns^ | 0.03^*^ | 0.04^*^ |
| Φ_Po_ | 0.05^*^ | 0.04^*^ | 0.4 ^ns^ |
| Φ_Eo_ | 0.03^*^ | 0.04^*^ | 0.2 ^ns^ |
| Φ_Do_ | 0.05^*^ | 0.04^*^ | 0.8^ns^ |
| Φ_PAV_ | 0.19 ^ns^ | 0.18 ^ns^ | 0.05^*^ |
| ψ_o_ | 0.49 ^ns^ | 0.25 ^ns^ | 0.05^*^ |
| NPQ | 0.38 ^ns^ | 0.12 ^ns^ | 0.01^**^ |
| Chl a | 0.01^**^ | 0.37 ^ns^ | 0.63 ^ns^ |
| Chl b | 0.02^*^ | 0.80 ^ns^ | 0.67 ^ns^ |
| Total Chl | 0.01^**^ | 0.65 ^ns^ | 0.68 ^ns^ |
| Carotenoids | 0.01^**^ | 0.50 ^ns^ | 0.73 ^ns^ |
| MDA | 0.01^**^ | 0.01^**^ | 0.15 ^ns^ |
| APX | 0.99 ^ns^ | 0.05^*^ | 0.56 ^ns^ |
| SOD | 0.10 ^ns^ | 0.01^**^ | 0.48 ^ns^ |
| CAT | 0.01^**^ | 0.01^**^ | 0.42 ^ns^ |
| H_2_O_2_ | 0.66 ^ns^ | 0.01^**^ | 0.83 ^ns^ |
| Soluble carbohydrates | 0.05^*^ | 0.01^**^ | 0.36 ^ns^ |
| Starch | 0.05^**^ | 0.01^**^ | 0.85 ^ns^ |

ns: Non significance. ^*^Significance at 0.05 probability level. ^**^ Significance at 0.01

**Figure S1.** Non-photochemical quenching (NPQ) derived from chlorophyll fluorescence parameters in the leaves of rose plants grown at different light spectrums [blue (B), red (R), white (W) and red and blue (RB)] under 250 (black bars) and 1500 (gray bars) µmol m^-2^ s^-1^ PPFD. Bars represent means ± SD.

**Figure S2.** Chlorophyll a (**A**), chlorophyll b (**B**) and total chlorophyll (**C**) concentrations in the leaves of rose plants grown at different light spectrums [blue (B), red (R), white (W) and red and blue (RB)] under 250 (black bars) and 1500 (gray bars) µmol m^-2^ s^-1^ PPFD. Bars represent means ± SD.
